# Use of ecological momentary assessment to detect variability in mood, sleep and stress in bipolar disorder

**DOI:** 10.1186/s13104-019-4834-7

**Published:** 2019-12-04

**Authors:** Han Li, Dahlia Mukherjee, Venkatesh Basappa Krishnamurthy, Caitlin Millett, Kelly A. Ryan, Lijun Zhang, Erika F. H. Saunders, Ming Wang

**Affiliations:** 1 Department of Public Health Science, Penn State College of Medicine, Penn State Milton S. Hershey Medical Center, 90 Hope Drive, Hershey, PA 17033 USA; 2 Department of Psychiatry, Penn State College of Medicine, Penn State Milton S. Hershey Medical Center, Hershey, PA USA; 3 Department of Psychiatry, University of Michigan Medical School, Ann Arbor, MI USA; 4 Institute of Personalized Medicine, Penn State College of Medicine, Penn State Milton S. Hershey Medical Center, Hershey, PA USA

**Keywords:** Multilevel models, Subject heterogeneity, Ecological Momentary Assessment, Mood disorders, Mania, Affective disorders

## Abstract

**Objective:**

Our aim was to study within-person variability in mood, cognition, energy, and impulsivity measured in an Ecological Momentary Assessment paradigm in bipolar disorder by using modern statistical techniques. Exploratory analyses tested the relationship between bipolar disorder symptoms and hours of sleep, and levels of pain, social and task-based stress. We report an analysis of data from a two-arm, parallel group study (bipolar disorder group N = 10 and healthy control group N = 10, with 70% completion rate of 14-day surveys). Surveys of bipolar disorder symptoms, social stressors and sleep hours were completed on a smartphone at unexpected times in an Ecological Momentary Assessment paradigm twice a day. Multi-level models adjusted for potential subject heterogeneity were adopted to test the difference between the bipolar disorder and health control groups.

**Results:**

Within-person variability of mood, energy, speed of thoughts, impulsivity, pain and perception of skill of tasks was significantly higher in the bipolar disorder group compared to health controls. Elevated bipolar disorder symptom domains in the evening were associated with reduced sleep time that night. Stressors were associated with worsening of bipolar disorder symptoms. Detection of symptoms when an individual is experiencing difficulty allows personalized, focused interventions.

## Introduction

Bipolar disorders (BD) are episodic, recurrent brain disorders characterized by manic (BD type I) or hypomanic (BD type II) episodes and depressive episodes causing disturbances in mood, motivation, hedonic capacity, activity levels, sleep, energy and judgment that impair functioning [1]. In the United States, approximately 1–4% of the population is affected by BD [2]. Diagnosis of BD, as with other psychiatric disorders, relies largely on self-report of symptoms [1, 3], however, it is difficult for individuals to recall mood symptoms, and recall may be influenced by current mood state [4].

Most people with BD suffer from subsyndromal symptoms between manic and depressive episodes [5]. The presence of subsyndromal symptoms has been shown to increase the likelihood of emergence of a mood episode [6–8], and variability in mood symptoms has been associated with deficits in emotional processing [9], and functional impairment [10, 11]. The temporal relationship between variables thought to affect mood such as sleep disturbance and psychosocial stress are important in determining the associated risk factors. Such patient-reported data on sleep and psychosocial stressors can help determine when to intervene to prevent the development of a mood episode [12–17]. However, in the literature, the relationship between sleep and mood is not clear. For instance, in several longitudinal studies, longer sleep onset latency, longer wakefulness after sleep onset and lower sleep efficiency have been associated with higher negative affect in BD [14]. However, an experimental design to alter evening mood prior to sleep showed that induction of happy mood prior to sleep onset created a shorter sleep onset latency in the HC group, but not in BD group, a phenomenon perhaps akin to what we see in the naturalistic setting [18].

Through technological advancements, daily self-reporting of mood and behavior is more easily recorded than in the past and provides valuable information on symptoms during daily life than traditional research methods [19]. Ecological Momentary Assessment (EMA) is a methodology allowing participants to report on phenomena close in time to when symptoms are experienced and to document several events or time periods, often in 1 day [16, 17, 20, 21]. In previous studies on EMA, technologies such as smart phones and computers have been used effectively to gather data in patients with BD [22–27]. In our prior study, we tested the feasibility of using EMA ratings collected for 14 days by smartphones with 70% completion rate of daily EMA surveys in BD and HC participants [28]. In the present work, we perform a secondary analysis using data from this prior study to conduct multi-level models adjusted for potential subject heterogeneity, and extend our findings.

## Main text

### Design, sample and measures

The research was approved by the Institutional Review Board (IRB) at the Hershey Medical Center (PSU COM IRB # 00251, Approval 3/28/2014). This study protocol was designed to test the compliance of individuals in a BD and an HC group on completion of twice-daily mood and stress, and once daily sleep measures on a Motorola Droid RazrM smartphone provided to the subjects for the study for 14 days. The feasibility outcomes of the study have been described previously [28]. The participants were screened over the phone, then seen in person for one visit. During that visit, the Mini-International Neuropsychiatric Interview (MINI), version 5.0 [29] was completed, demographic data collected and the participants were instructed on the use of the smartphone.

The twice-daily mood and stress survey measures included single item questions rating mood, energy, speed of thoughts, impulsivity, and physical pain on a visual analogue scale. Mood was described to the participants as being positive/negative valance of subjective experience. Note that the measurement of mood is of the subjective experience and not of a diagnosed mood episode. Two questions assessing the current and preferred social situation (alone/with others) were assessed along a Likert scale. Task-based stress was measured through three questions assessing perception of skill, effort and preference of tasks on a Likert scale. Once daily, in the morning, the participants entered the time to bed the previous night, time to sleep the previous night, number of awakenings, final time of waking, and time out of bed. More details about the enrollment criteria and data structure can be referred to Additional file 1.

### Statistical analysis

#### Within-subject variability in BD symptoms

We tested the primary hypothesis that within-subject variability in the core BD symptoms of mood valance, speed of thoughts, energy and impulsivity was greater in the BD group than in the HC group. First, we calculated the intraclass correlation coefficient (ICC) for each group to examine the relative magnitude of between-person versus within-person variation. Then, we tested the level-1 heterogeneity of variance with the null hypothesis of no individual differences in within-person variation [30, 31]. Lastly, multilevel models were further applied to analyze the variation in BD symptoms and stressors between the BD and HC groups [32].

#### Evaluation of the associations between BD symptoms and sleep time

To analyze the associations between core BD symptoms and sleep time, we answered the following questions: (I) are the core BD symptoms affected by prior nights’ sleep duration, and does the effect last more than 1 day? (II) Are core BD symptoms during the day affecting that night’s total sleep time, and does this effect last more than one night? Two analyses using the morning and evening measures of the core BD symptoms were conducted with details shown in Additional file 1. Similar multilevel models described above were fitted with the variables of age, gender, employment status, time (days), group (1 = BD; 0 = HC), sleep time/BD symptoms as well as the interactions between group and sleep time/BD symptom (if not significant, excluded for the final model) included as fixed effects.

#### Evaluation of effects of pain and social stress on BD symptoms

To analyze the association between pain or social and task-based stressors (i.e., social stress, perception of skill) and core BD symptoms, we adopted similar models above with BD symptoms as longitudinal outcomes, and the pain or social stressors were included as time-varying covariates with fixed effects. The other variables including group (1 = BD; 0 = HC), age, gender, employment status and time (days) as well as the interaction of group and the pain or social stressors (if not significant, were excluded for the final model) were also considered as fixed effects.

The strategies to handle small sample size are provided in Additional file 1. All hypotheses tests are two-sided with the significance level of 0.05. Data was analyzed using SAS 9.4 Software with the MIXED Procedure.

### Results

All BD participants were diagnosed with BD, type I. The groups did not differ significantly based on age, gender and employment status (p > 0.05).

### Within-subject variability in BD symptoms (Table 1)

Table 1 summarizes the analysis of distribution and difference in within-person and between-person variability between the BD group and HC group in BD core symptoms, stressors and pain (i.e., the ICC for mood in the BD group is 0.55, indicating 55% between-person variation and 45% within-person variability). For multilevel models, no significant fixed effects for time were found. Overall, higher within-person variabilities in core BD symptoms as well as pain and perception of skill were shown in the BD group compared to the HC group (Table 1). There were no group differences in the mean scores of speed of thoughts, impulsivity, social stress, perception of skill, effort or task preference. The significant results above remained same after correction for multiple testing, thus original p-values are presented.

**Table 1:** Variability of BD symptoms and stressors between BD and HC groups over the 14-day period

	ICC	Estimates of group effect on within-person variability (*α*_1_)	p-value for group effect on within-person variability^a^	Estimates of between-group difference the mean level of symptoms or stressors (*γ*_01_)	p-value for between-group difference in the mean level of symptoms or stressors^b^
Mood					
BD	0.55	0.86	< *0.0001*	− 18.42	*0.015*
HC	0.72				
Energy					
BD	0.49	1.02	< *0.0001*	− 20.99	*0.006*
HC	0.61				
Speed of thoughts					
BD	0.40	1.73	< *0.0001*	− 0.10	0.99
HC	0.67				
Impulsivity					
BD	0.16	0.89	< *0.0001*	13.42	0.07
HC	0.68				
Pain					
BD	0.80	1.64	< *0.0001*	38.75	*0.001*
HC	0.40				
Social stress					
BD	0.20	0.03	0.85	0.61	0.06
HC	0.02				
Perception of skill					
BD	0.23	0.65	< *0.0001*	0.84	0.05
HC	0.54				
Effort					
BD	0.12	0.10	0.50	0.28	0.53
HC	0.23				
Preference of tasks					
BD	0.13	0.06	0.68	0.48	0.17
HC	0.11				
Sleep (h)					
BD	0.46	0.73	< *0.0001*	− 0.67	0.27
HC	0.30				

*ICC* intraclass correlation coefficient

^a^The p-values for group effect on within-person variability

^b^The p-values for between-group difference in the mean level of symptoms or stressors. Significant results are in italic

### Evaluation of the associations between BD symptoms and sleep time (Table 2)

The effect of the prior hours of sleep on BD symptoms in the morning of the index day across 13 days were examined with no significant results (Additional file 1: Table S1, p > 0.05). Also, we tested the effect of evening symptoms on total hours of sleep for the same night (Model 1) and the same night plus the subsequent night (Model 2). Evening mood symptoms had a negative relationship to sleep, indicating elevated mood was associated with a decrease in sleep hours in the subsequent night (p = 0.03). The same effect was found for energy (p = 0.04). Elevated speed of speed of thoughts and impulsivity were associated with decreased sleep for the next night only (p < 0.05).

**Table 2:** The associations between core BD evening symptoms during/before each day and that night’s sleep time

Variable	Final selected model		
	Estimate	SE	p-value
BD	− 1.79	0.84	*0.04*
T mood	− 0.01	0.01	0.16
T_−1_ mood	− 0.02	0.01	*0.03*
BD	− 0.31	0.61	0.62
T speed of thoughts	− 0.02	0.01	*0.01*
T_−1_ speed of thoughts	–	–	–
BD	− 1.78	0.90	0.07
T energy	− 0.02	0.01	*0.01*
T_−1_ energy	− 0.01	0.01	*0.04*
BD	− 0.25	0.66	0.70
T impulsivity	− 0.01	0.01	*0.049*
T_−1_ impulsivity	–	–	–

Model 1 includes the daily core BD evening symptoms during each index day (T); Model 2 includes both the daily core BD evening symptoms during the index day and the day before the index day (T_−1_). Both models are final selected models from two candidate models, which are adjusted for age, gender, employment status, time (days) and the interactions between BD group (not included if not significant). Significant results are in italic

### Evaluation of effects of pain and social stress on BD symptoms (Table 3)

We evaluated the effect of pain, social stress, perception of skill, effort and task-preference on mood symptoms at the same time point. Mood (Table 3A) was associated inversely with pain, and positively with task preference; mood was not associated with social stress, task effort or skill. Elevated energy (Table 3B) was associated with lower perceived skill, however, elevated energy was also associated with effort, indicating that greater effort on a task and greater energy were associated in time. Energy was not associated with pain, task preference or social stress. Increased speed of thoughts (Table 3C) was associated with lower perceived skill, but not with pain, task effort, task preference or social stress. Greater impulsivity (Table 3D) was associated with less pain, lower perception of skill, and higher social stress in the BD group, but not associated with task effort or task preference.

**Table 3:** The association of pain or social stressors with core BD symptoms at the same timepoint

A. Mood	Estimate	SE	p-value	B. Energy	Estimate	SE	p-value
BD	− 15.82	7.30	*0.04*	BD	− 12.72	7.27	0.09
Pain	− 0.12	0.05	*0.01*	Pain	0.06	0.15	0.67
BD * pain				BD * pain	− 0.30	0.16	0.06
BD	− 20.06	7.11	*0.01*	BD	− 23.19	6.93	*0.003*
Skill	− 0.97	0.57	0.09	Skill	− 1.48	0.66	*0.03*
BD * skill				BD * skill			
BD	− 20.94	7.10	*0.01*	BD	− 25.03	6.86	*0.002*
Effort	0.07	0.33	0.82	Effort	1.29	0.37	*0.001*
BD * effort				BD * effort			
BD	− 30.03	7.73	*0.001*	BD	− 24.63	6.97	*0.002*
Preference	− 0.51	0.53	0.34	Preference	0.29	0.42	0.50
BD * preference	2.19	0.72	*0.003*	BD * preference			
BD	− 20.91	7.10	*0.01*	BD	− 24.39	6.97	*0.002*
Social stress	− 0.005	0.40	0.99	Social stress	− 0.10	0.47	0.82
BD * social stress				BD * social stress			

C. Speed of thoughts	Estimate	SE	p-value	D. Impulsivity	Estimate	SE	p-value
BD	3.45	7.46	0.65	BD	16.62	7.97	*0.048*
Pain	− 0.10	0.06	0.10	Pain	0.68	0.19	< *0.001*
BD * pain				BD * pain	− 0.72	0.20	< *0.001*
BD	0.62	6.69	0.93	BD	21.11	7.97	*0.01*
Skill	− 1.49	0.71	*0.04*	Skill	2.68	1.46	0.07
BD * skill				BD * skill	− 4.66	1.77	*0.01*
BD	− 1.01	6.51	0.88	BD	11.27	7.37	0.14
Effort	0.77	0.41	0.06	Effort	− 0.29	0.48	0.55
BD * effort				BD * effort			
BD	− 0.74	6.60	0.91	BD	11.42	7.36	0.14
Preference	0.08	0.46	0.85	Preference	− 0.40	0.54	0.46
BD * preference				BD * preference			
BD	− 0.84	6.60	0.90	BD	1.62	8.12	0.84
Social stress	0.27	0.50	0.59	Social stress	− 2.20	0.86	*0.01*
BD * social stress				BD * social stress	3.18	1.17	*0.007*

A. mood, B. energy, C. speed of thoughts, D. impulsivity. Thoughts: speed of thoughts; Skill: perception of skill; Preference: preference of tasks. All models are adjusted for age, adjusted for age, gender, employment status, time (days). Significant results are in italic

## Discussion

In this analysis, we illustrated how extensions of multilevel models could be used to analyze EMA for valid and informative inference, and found significantly elevated within-person variability in core BD symptoms including mood, energy, speed of thoughts, and impulsivity in the BD group when compared to the HC group. The EMA method allows for gathering the daily variability of mood and related symptoms in BD [25–27, 33–37]. Traditional methods of assessment ask participants to retrospectively reflect upon mood symptoms over a period of time, and do not always reflect the instability of mood and functioning, which is disruptive to patients. Our study highlights that not only absolute differences in mood states, but also within-subject mood variation over time, can be captured using EMA techniques in patients with BD.

This study results allowed us to look at the effect of self-reported hours of sleep on symptoms the next day. We found that the total hours of sleep reported did not affect symptoms the following day in BD subjects, elevated evening mood, energy and impulsivity were associated with reduced sleep on that night. Our study used a different scale to measure mood, and thus may have captured different facets of this core symptom. Additionally, we measured mood only subjectively and did not have an objective measure that can include physical signs of affect.

We also found that low mood was associated with pain, and elevated mood was associated with more enjoyment of tasks. Increased speed of thoughts and elevated energy were associated with lower perceived skill, which may indicate impairment, though we did not measure impairment directly. Impulsivity was associated with less pain, lower perception of skill and higher social stress in the BD group. Proximal stressful life events have been shown to negatively affect sleep in inter-episode BD [38], and responses to negative events were more stressful for those with BD than HC, which was associated with higher cortisol levels [18, 39]. Detection of changes related to stressors through smartphone technology between office visits or encounters with the health care team may allow for the opportunity to intervene and prevent mood episodes.

Through the use of EMA, we detected daily variability in BD symptoms and associations between daily mood, energy, and impulsivity symptoms in BD, sleep and daily stressors. Further exploration of the proximal relationship between daily stressors, sleep and mood is needed.

## Limitations

There are some limitations to this study. This study was designed to demonstrate feasibility; therefore, the sample size was small. In future studies, we will extend our study period in a larger sample, which is ongoing now. This report does not address the psychometric properties of the items. Also, certain biases and confounding factors are still present when using smart phones to capture data, which needs more exploration.

## Supplementary information

**Additional file 1.** More detailed information on study enrollment, data structure and statistical analysis.

## Data Availability

The datasets supporting the conclusions of this article is available based upon reasonable request.

## Abbreviations

EMA: Ecological Momentary Assessment
BD: bipolar disorder
HC: healthy control
IRB: Institutional review board
MINI 5.0: Mini-International Neuropsychiatric Interview, version 5.0
DREAM: Dynamic Real-Time Ecological Ambulatory Methodologies
