# Supplementary material for: Use of ecological momentary assessment to detect variability in mood, sleep and stress in bipolar disorder
Source: BMC Res Notes. 2019 Dec 4;12:791. doi: 10.1186/s13104-019-4834-7 (PMC6894147; doi:10.1186/s13104-019-4834-7)
Supplement: Supplementary file 1 — Additional file 1. More detailed information on study enrollment, data structure and statistical analysis. [file 13104_2019_4834_MOESM1_ESM.docx]

Additional file for “Use of Ecological Momentary Assessment to Detect Variability in Mood, Sleep and Stress in Bipolar Disorder”

Han Li,^a^ Dahlia Mukherjee,^b^ Venkatesh Basappa Krishnamurthy,^b^ Caitlin Millett,^b^

Kelly A. Ryan,^c^ Lijun Zhang, ^d^ Erika F.H. Saunders,^b^* Ming Wang ^a^*

^a^ Department of Public Health Science, Penn State College of Medicine, Hershey, PA, United States

^b^ Department of Psychiatry, Penn State College of Medicine, Penn State Milton S. Hershey Medical Center, Hershey, PA, United States

^c^ University of Michigan Medical School, Department of Psychiatry, Ann Arbor, MI, United States

^d^ Institute of Personalized Medicine, Penn State College of Medicine, Penn State Milton S. Hershey Medical Center, Hershey, PA, United States

*Denoting co-senior authorship

Author e-mail addresses:

Han Li [LIhan2015@hotmail.com](mailto:LIhan2015@hotmail.com)

Dahlia Mukherjee [dmukherjee@pennstatehealth.psu.edu](mailto:dmukherjee@pennstatehealth.psu.edu)

Basappa Krishnamurthy, Venkatesh [vbasappakrishnamurth@pennstatehealth.psu.edu](mailto:vbasappakrishnamurth@pennstatehealth.psu.edu)

Caitlin Millett [cmillett@pennstatehealth.psu.edu](mailto:cmillett@pennstatehealth.psu.edu)

Kelly Ryan [karyan@med.umich.edu](mailto:karyan@med.umich.edu)

Lijun Zhang [lzhang6@pennstatehealth.psu.edu](mailto:lzhang6@pennstatehealth.psu.edu)

Erika F.H. Saunders [esaunders@pennstatehealth.psu.edu](mailto:esaunders@pennstatehealth.psu.edu)

Correspondence concerning this article should be addressed to

Ming Wang, PhD

Associate Professor in Biostatistics

Department of Public Health Sciences, College of Medicine

Penn State Milton S. Hershey Medical Center

90 Hope Drive, Hershey, PA 17033

Contact: [mwang@phs.psu.edu](mailto:mwang@phs.psu.edu)

**Inclusion and exclusion criteria**

Subjects were included in the BD group if they were a minimum age of 18 and had a diagnosis of either BD I or II by the Mini-International Neuropsychiatric Interview, version 5.0 (MINI 5.0) [[1](#_ENREF_1)]. Subjects were included in the HC group if they were a minimum age of 18 and had no family history of any major psychiatric illness, and no personal history of major mood, anxiety or psychotic psychiatric illness, as determined by the MINI 5.0. The exclusion criteria for both groups included the inability to use smartphones or read English.

Smartphones were programmed by the Survey Research Center at Penn State University Park Dynamic Real-Time Ecological Ambulatory Methodologies group. All other features of the phone were disabled. Data were collected on a secure ITS-hosted virtual server running Windows Server 2008, and transmitted to the server and stored in a local MySQL database. All data were protected through encryption.

**Data Structure**

The mood and stress surveys were auto-generated by the smartphone and prompted participants to complete data collection at random times twice per day for 14 days. The first survey was generated during a 4-hour time-period in the morning, and the second survey was generated during a 4-hour time-period in the evening. To ensure that the notification of the survey did not wake the subjects, during the initial visit the subjects chose between 4 schedules for the 4-hour windows based on usual sleep habits: Very Early, Early, Late and Very Late. Each participant was instructed at the start of the study to complete all EMA surveys. Data including demographics, BD core symptoms (mood, speed of thoughts, impulsivity, energy), stressors and total sleep time (hours) were analyzed. Mood, speed of thoughts, impulsivity, energy, and physical pain were measured on a visual analogue scale. The data from these four questions were converted from the visual analogue scale to a 0-100 measurement.

Social stress and task-based stress were measured based on the work of Myin-Germeys [[2](#_ENREF_2)]. The measure of social stress included two statements: “I am with others” (dichotomous response yes/no) and “I would rather be alone,” (7-point Likert Scale). The Likert scale was coded 1= “Strongly Agree” and 7= “Strongly Disagree,” if the response to “I am with others” was “no,” and was reverse-coded (1= “Strongly Disagree, 7= “Strongly Agree”) if the response was “yes.” Using this scoring method, incongruence between current social state and desired social state was rated a “7,” and congruence was rated as “1.” ​ Task-based stress was measured with three statements that assessed skill “I am skilled to do this activity,” “This activity does not require effort,” and preference “I would rather be doing this than something else” based on a Likert Scale (1= “Strongly Agree” and 7= “Strongly Disagree). The description of the task was not captured; the question focused on the perception of the task. Demographic information including age, gender and employment status were included as potential confounders for analysis.

**Statistical Analysis**

**A.** Note that the ICC was calculated for each group based on unconditional models. The multilevel models were applied to analyze the variation in BD symptoms and stressors between the BD and HC groups, by incorporating potential individual difference across group in within-person variation and a random intercept accounting for between-person heterogeneity in each symptom or stressor. The model is as follows:

Level 1: $y_{id}=\beta_{0id}+e_{id}$

Level 1 (within-person variability): $\sigma_{i}^{2}=\alpha_{0}*(\exp\left( \alpha_{1}*{group}_{i} \right))$

Level 2 (between-person variability): $\beta_{0id}=\gamma_{00}+\gamma_{01}*{group}_{i}+\gamma_{02}*{time}_{id}+U_{0i}$

where the Level 1 model described variation across days and the Level 2 model described variation between persons with the fixed effects of group (1=BD; 0=HC), time (days) and other variables including age, gender and employment status. In Level 1, $y_{\mathrm{id}}$ was the observed BD symptoms or stressors score for individual i on day d; $e_{id}$was the residual term following up a normal distribution $N\left( 0, \sigma_{i}^{2} \right)$. If the level-1 heterogeneity of variance was detected, then $\sigma_{i}^{2}$ was modelled to vary over individuals by group (see Level 1); otherwise, $\sigma_{i}^{2}=\sigma^{2}$ indicated homogenous within-person variation. In Level 2, $\beta_{0id}$ was the expected mean level for individual i on day d; $\gamma_{00}$ was the expected mean level for HC subjects at the baseline (day 0); $\gamma_{01}$ represented the difference on expected mean level for BD subjects compared to HC subjects across days, and $\gamma_{02}$ showed the temporal effect on the mean level;$U_{0i}$ was the random intercept to account for between-person variation, which followed up $N\left( 0, \sigma_{u}^{2} \right)$ and was assumed to be independent with $e_{id}.$The BD symptoms and stressors were measured over a 14-day period. Note that a Bonferroni correction was performed for adjusted p-values.

**B.** The analysis plan to evaluate the associations between BD symptoms and sleep time is as follows: (I) For analysis I, we chose the morning measures as the daily values for the core BD symptoms on an index day (T), in which each day across 13 days becomes an index day. The predictors related to sleep time include the prior night’s sleep time (T_-1_), the day before the prior night’s sleep time (T_-2_) and the day before the prior two-night’s sleep time (T_-3_); (II) For analysis II, we chose the evening measures as the daily values for the core BD symptoms on each index day (T) across the 14-day period. Thus, the sleep times across 14 days are treated as a longitudinal outcome with the daily core BD symptoms during that day (T) and the day before (T_-1_) as the predictors.

**C.** Due to the small sample size, all the models were estimated on the combined person-period data set [[3](#_ENREF_3)], and several strategies were considered for parameter estimation and statistical inferences. For instance, the estimates for fixed effects were based maximum likelihood, and for the parameters in residuals and random effects, restricted maximum likelihood was applied [[4](#_ENREF_4), [5](#_ENREF_5)]. In addition, the significance of fixed effects was evaluated with Wald’s tests with the Satterthwaite denominator degrees of freedom, which is recommended in the presence of small sample size [[5-7](#_ENREF_5)].

**Table S1.** The associations between prior night’s sleep time and BD morning symptoms on index day (T)

|  | Model 1 | | | Model 2 | | |
| --- | --- | --- | --- | --- | --- | --- |
|  | Estimate | SE | P-value | Estimate | SE | P-value |
| **Mood** |  |  |  |  |  |  |
| Intercept | 27.16 | 19.49 | 0.18 | 23.04 | 20.29 | 0.27 |
| BD | -21.90 | 6.20 | 0.003 | -23.27 | 6.09 | 0.002 |
| Age | 0.73 | 0.29 | 0.02 | 0.79 | 0.29 | 0.01 |
| Gender (=female) | -7.55 | 7.52 | 0.33 | -8.94 | 7.39 | 0.25 |
| Employment | 18.28 | 9.93 | 0.08 | 20.36 | 9.74 | 0.05 |
| Time (days) | -0.19 | 0.21 | 0.36 | -0.01 | 0.23 | 0.96 |
| T_-1_ Sleep | 0.21 | 0.64 | 0.74 | 0.17 | 0.64 | 0.79 |
| T_-2_ Sleep | -0.80 | 0.61 | 0.19 | -1.04 | 0.67 | 0.12 |
| T_-3_ Sleep |  |  |  | 0.25 | 0.64 | 0.69 |
| **Energy** |  |  |  |  |  |  |
| Intercept | 15.54 | 20.50 | 0.46 | 14.13 | 22.02 | 0.53 |
| BD | -21.79 | 6.38 | 0.004 | -23.87 | 6.44 | 0.002 |
| Age | 0.69 | 0.29 | 0.03 | 0.73 | 0.30 | 0.03 |
| Gender (=female) | -8.27 | 7.75 | 0.30 | -7.51 | 7.83 | 0.35 |
| Employment | 23.33 | 10.22 | 0.04 | 25.49 | 10.29 | 0.03 |
| Time (days) | -0.54 | 0.24 | 0.03 | -0.50 | 0.28 | 0.07 |
| T_-1_ Sleep | 0.36 | 0.73 | 0.62 | 0.46 | 0.75 | 0.54 |
| T_-2_ Sleep | 0.59 | 0.70 | 0.40 | 0.34 | 0.78 | 0.67 |
| T_-3_ Sleep |  |  |  | -0.19 | 0.74 | 0.79 |
| **Speed of thoughts** |  |  |  |  |  |  |
| Intercept | 27.16 | 21.42 | 0.22 | 40.29 | 22.18 | 0.08 |
| BD | -2.13 | 6.92 | 076 | -3.28 | 6.74 | 0.63 |
| Age | 0.23 | 0.32 | 0.47 | 0.19 | 0.31 | 0.56 |
| Gender (=female) | 9.54 | 8.39 | 0.27 | 10.31 | 8.18 | 0.23 |
| Employment | 3.70 | 11.10 | 0.74 | 3.62 | 10.77 | 0.74 |
| Time (days) | -0.10 | 0.20 | 0.60 | -0.22 | 0.23 | 0.35 |
| T_-1_ Sleep | 0.09 | 0.66 | 0.89 | -0.10 | 0.70 | 0.89 |
| T_-2_ Sleep | 0.22 | 0.61 | 0.72 | -0.20 | 0.71 | 0.78 |
| T_-3_ Sleep |  |  |  | -0.86 | 0.65 | 0.19 |
| **Impulsivity** |  |  |  |  |  |  |
| Intercept | -23.34 | 22.81 | 0.32 | -5.44 | 24.55 | 0.83 |
| BD | 10.83 | 7.10 | 0.15 | 9.89 | 7.14 | 0.19 |
| Age | 0.67 | 0.33 | 0.06 | 0.55 | 0.33 | 0.12 |
| Gender (=female) | -2.48 | 8.63 | 0.78 | -0.65 | 8.68 | 0.94 |
| Employment | 23.52 | 11.38 | 0.06 | 21.75 | 11.41 | 0.08 |
| Time (days) | 0.27 | 0.26 | 0.31 | 0.14 | 0.30 | 0.65 |
| T_-1_ Sleep | 0.72 | 0.82 | 0.39 | 0.89 | 0.86 | 0.30 |
| T_-2_ Sleep | -0.97 | 0.77 | 0.21 | -1.23 | 0.89 | 0.17 |
| T_-3_ Sleep |  |  |  | -1.53 | 0.83 | 0.07 |

Model 1 includes both the prior night’s total sleep time (denoted by “T_-1_ Sleep”) and the day before the prior night’s total sleep time (denoted by “T_-2_ Sleep”); Model 2 includes the prior night’s total sleep time (denoted by “T_-1_ Sleep”), the day before the prior night’s total sleep time (denoted by “T_-2_ Sleep”) and the day before the prior two-night’s sleep time (denoted by “T_-3_ Sleep”). Both models are adjusted for age, gender, employment status, time (days) and the interactions are not considered if not significant. Significant results are in bold.

**Table S2.** The association between core BD evening symptoms during/before each day and that night’s sleep time

|  | Model 1 | | | Model 2 | | |
| --- | --- | --- | --- | --- | --- | --- |
|  | Estimate | SE | P-value | Estimate | SE | P-value |
| **Sleep hour** |  |  |  |  |  |  |
| Intercept | 9.67 | 1.91 | 0.001 | 10.68 | 2.38 | 0.001 |
| BD | -0.63 | 0.70 | 0.38 | -1.79 | 0.84 | 0.04 |
| Age | -0.03 | 0.03 | 0.39 | -0.01 | 0.04 | 0.79 |
| Gender (=female) | -0.14 | 0.81 | 0.86 | -0.88 | 1.05 | 0.42 |
| Employment | -0.68 | 1.08 | 0.54 | 0.15 | 1.21 | 0.90 |
| Time (days) | -0.02 | 0.03 | 0.55 | 0.01 | 0.04 | 0.74 |
| T Mood | -0.01 | 0.01 | 0.21 | -0.01 | 0.01 | 0.16 |
| T_-1_ Mood |  |  |  | -0.02 | 0.01 | 0.03 |
| **Sleep hour** |  |  |  |  |  |  |
| Intercept | 1037 | 1.76 | <0.001 | 12.08 | 1.99 | <0.001 |
| BD | -0.31 | 0.61 | 0.62 | -0.72 | 0.62 | 0.27 |
| Age | -0.04 | 0.03 | 0.23 | -0.04 | 0.03 | 0.19 |
| Gender (=female) | 0.20 | 0.74 | 0.79 | -0.46 | 0.83 | 0.59 |
| Employment | -0.97 | 0.97 | 0.33 | -0.80 | 0.95 | 0.42 |
| Time (days) | -0.02 | 0.03 | 0.41 | 0.02 | 0.04 | 0.66 |
| T Speed of thoughts | -0.02 | 0.01 | 0.01 | -0.03 | 0.01 | 0.001 |
| T_-1_ Speed of thoughts |  |  |  | -0.01 | 0.01 | 0.19 |
| **Sleep hour** |  |  |  |  |  |  |
| Intercept | 9.62 | 1.94 | <0.001 | 10.78 | 2.65 | 0.002 |
| BD | -0.57 | 0.70 | 0.43 | -1.78 | 0.89 | 0.07 |
| Age | -0.03 | 0.03 | 0.36 | -0.01 | 0.04 | 0.78 |
| Gender (=female) | -0.17 | 0.83 | 0.84 | -1.31 | 1.17 | 0.29 |
| Employment | -0.71 | 1.09 | 0.53 | 0.24 | 1.33 | 0.86 |
| Time (days) | -0.02 | 0.03 | 0.46 | 0.02 | 0.04 | 0.62 |
| T Energy | -0.01 | 0.01 | 0.22 | -0.02 | 0.01 | 0.01 |
| T_-1_ Energy |  |  |  | -0.01 | 0.01 | 0.04 |
| **Sleep hour** |  |  |  |  |  |  |
| Intercept | 9.37 | 1.85 | 0.001 | 9.79 | 2.30 | 0.001 |
| BD | -0.25 | 0.66 | 0.70 | -0.57 | 0.75 | 0.46 |
| Age | -0.03 | 0.03 | 0.34 | -0.03 | 0.04 | 0.45 |
| Gender (=female) | -0.15 | 0.79 | 0.85 | -0.62 | 1.01 | 0.55 |
| Employment | -0.65 | 1.04 | 0.54 | -0.36 | 1.16 | 0.76 |
| Time (days) | -0.02 | 0.03 | 0.39 | -0.01 | 0.04 | 0.85 |
| T Impulsivity | -0.01 | 0.001 | 0.049 | -0.01 | 0.01 | 0.16 |
| T_-1_ Impulsivity |  |  |  | -0.01 | 0.01 | 0.36 |

Model 1 includes the daily core BD evening symptoms during each index day (T); Model 2 includes both the daily core BD evening symptoms during the index day and the day before the index day (T_-1_). Both models are adjusted for age, gender, employment status, time (days) and the interactions between BD group (not included if not significant). Significant results are in bold.

**References:**

1. Sheehan DV, Lecrubier Y, Sheehan KH, Amorim P, Janavs J, Weiller E, Hergueta T, Baker R, Dunbar GC: **The Mini-International Neuropsychiatric Interview (M.I.N.I.): the development and validation of a structured diagnostic psychiatric interview for DSM-IV and ICD-10**. *The Journal of clinical psychiatry* 1998, **59 Suppl 20**:22-33;quiz 34-57.

2. Myin-Germeys I, Peeters F, Havermans R, Nicolson NA, DeVries MW, Delespaul P, Van Os J: **Emotional reactivity to daily life stress in psychosis and affective disorder: an experience sampling study**. *Acta psychiatrica Scandinavica* 2003, **107**(2):124-131.

3. Hoffman L, Rovine MJ: **Multilevel models for the experimental psychologist: foundations and illustrative examples**. *Behav Res Methods* 2007, **39**(1):101-117.

4. Raudenbush SW, Bryk AS: **Hierarchical linear models : applications and data analysis methods**, 2nd edn. California: Sage Publications; 2002.

5. Fitzmaurice GM, Laird NM, Ware JH: **Applied longitudinal analysis**, 2nd edn. Hoboken, N.J.: Wiley; 2011.

6. Hox JJ, Roberts JK: **Handbook of Advanced Multilevel Analysis Preface**. *Eur Assoc Methodol S* 2010:Vii-Viii.

7. Browne WJ, Draper D: **Implementation and performance issues in the Bayesian and likelihood fitting of multilevel models**. *Computation Stat* 2000, **15**(3):391-420.
